# Neuropathology of Aging in Cats and its Similarities to Human Alzheimer’s Disease

**DOI:** 10.3389/fragi.2021.684607

**Published:** 2021-06-07

**Authors:** Lorena Sordo, Alessandra C. Martini, E. Fiona Houston, Elizabeth Head, Danièlle Gunn-Moore

**Affiliations:** ^1^ The Royal (Dick) School of Veterinary Studies and The Roslin Institute, The University of Edinburgh, Edinburgh, United Kingdom; ^2^ Department of Pathology and Laboratory Medicine, University of California, Irvine, Irvine, CA, United States

**Keywords:** neuropathology, aging, Alzheimer’s disease, cats, model, b-amyloid, tau, CDS

## Abstract

Elderly cats develop age-related behavioral and neuropathological changes that ultimately lead to cognitive dysfunction syndrome (CDS). These neuropathologies share similarities to those seen in the brains of humans with Alzheimer’s disease (AD), including the extracellular accumulation of *ß*-amyloid (Aβ) and intraneuronal deposits of hyperphosphorylated tau, which are considered to be the two major hallmarks of AD. The present study assessed the presence and distribution of Aβ and tau hyperphosphorylation within the cat brain (*n* = 55 cats), and how the distribution of these proteins changes with age and the presence of CDS. For this, immunohistochemistry was performed on seven brain regions from cats of various ages, with and without CDS (*n* = 10 with CDS). Cats accumulate both intracytoplasmic and extracellular deposits of Aβ, as well as intranuclear and intracytoplasmic hyperphosphorylated tau deposits. Large extracellular aggregates of Aβ were found in elderly cats, mainly in the cortical brain areas, with occasional hippocampal aggregates. This may suggest that these aggregates start in cortical areas and later progress to the hippocampus. While Aβ senile plaques in people with AD have a dense core, extracellular Aβ deposits in cats exhibited a diffuse pattern, similar to the early stages of plaque pathogenesis. Intraneuronal Aβ deposits were also observed, occurring predominantly in cortical brain regions of younger cats, while older cats had few to no intraneuronal Aβ deposits, especially when extracellular aggregates were abundant. Intracytoplasmic hyperphosphorylated tau was found within neurons in the brains of elderly cats, particularly in those with CDS. Due to their ultrastructural features, these deposits are considered to be pre-tangles, which are an early stage of the neurofibrillary tangles seen in AD. The largest numbers of pre-tangles are found mainly in the cerebral cortex of elderly cats, whereas lower numbers were found in other regions (i.e., entorhinal cortex and hippocampus). For the first time, intranuclear tau was found in both phosphorylated and non-phosphorylated states within neurons in the cat brain. The highest numbers of intranuclear deposits were found in the cortex of younger cats, and this tended to decrease with age. In contrast, elderly cats with pre-tangles had only occasional or no nuclear labelling.

## Introduction

In humans, age-related brain changes, such as brain atrophy, neuronal loss, and vascular pathology, may ultimately lead to dementia and Alzheimer’s disease (AD) (Raz, 2004; Jack et al., 2005; Glabe, 2006). The abnormal deposition of senile plaques containing *ß*-amyloid (Aβ) and neurofibrillary tangles (NFT) composed of hyperphosphorylated tau are major pathognomonic neuropathologies of AD, and are thought to play a key role in its development (Hardy and Allsop, 1991; Thal et al., 2002; Perl, 2010).

Similarly, aged cats develop age-related cognitive decline and eventually dementia, which is known as cognitive dysfunction syndrome (CDS; feline dementia) (Gunn-Moore et al., 2007; Landsberg et al., 2010). While the neuropathology of this condition is still poorly understood, several age-related neurodegenerative changes (i.e., brain atrophy, neuronal loss, vascular changes, and abnormal protein deposition) are found in the brains of elderly cats (Head et al., 2005; Gunn-Moore et al., 2006).

*β*-amyloid is generated from the cleavage of the amyloid precursor protein (APP) by *β* and *γ*-secretases. Since cleavage occurs at different sites, various lengths of Aβ can be formed, with Aβ40 and Aβ42 being found most commonly in the brain of patients with AD. While Aβ40 is the most abundant and tends to accumulate within blood vessels, Aβ42 tends to accumulate in the extracellular space and form the characteristic senile plaques of AD (Hardy and Allsop, 1991; Perl, 2010).

Diffuse Aβ aggregates are found in the brains of cats over 10 years of age (Cummings et al., 1996; Nakamura et al., 1996; Brellou et al., 2005; Head et al., 2005; Gunn-Moore et al., 2006; Gunn-Moore et al., 2007). Since these deposits do not have dense cores and share similarities to the diffuse deposits seen in non-demented humans, it has been suggested that the Aβ deposits seen in cats are less mature than the characteristic senile plaques of AD (Cummings et al., 1996; Nakamura et al., 1996; Colle et al., 2000; Head et al., 2000; Gunn-Moore et al., 2007). Similarly to human Aβ, deposits in cats are predominantly formed from the Aβ42 peptide (Cummings et al., 1996; Head et al., 2005). The lack of Aβ40 in cats may be due to its higher solubility, which promotes its rapid clearance, impeding its accumulation within the brain (Hyman et al., 1993; Cummings et al., 1996; Wisniewski et al., 1996). However, the Aβ40 peptide is found in association with cerebral blood vessels walls in cats, termed cerebral amyloid angiopathy (Nakamura et al., 1996). Both the pattern and distribution of Aβ in cats are more similar to those found in the elderly, non-demented human brain rather than patients with AD (Cummings et al., 1996; Nakamura et al., 1996; Brellou et al., 2005; Head et al., 2005; Gunn-Moore et al., 2007).

Tau is a microtubule-associated protein located in the cytosol of the cells and provides microtubule stability (Lee et al., 2005; Kametani and Hasegawa, 2018). When abnormally hyperphosphorylated, tau aggregates into paired helical filaments (PHF) that subsequently form NFT (Goedert et al., 1989; Hardy and Allsop, 1991; Lee et al., 2005; Perl, 2010).

Cats produce similar tau isoforms to those seen in humans (Chambers et al., 2015); however, there is still controversy regarding the presence of NFT in the brains of elderly cats. While some research found no evidence of NFT (Nakamura et al., 1996; Kuroki et al., 1997), others have reported intracytoplasmic hyperphosphorylated immunolabelling within neurons, which is believed to be an early stage of NFT, known as pre-tangles (Head et al., 2005; Gunn-Moore et al., 2006; Chambers et al., 2015).

The aim of this study was to assess the presence and distribution of Aβ and tau pathology in the brains of cats of various ages, with and without CDS. It was hypothesized that elderly cats would have larger and more frequent extracellular Aβ deposits and higher numbers of pre-tangles than younger cats. It was also hypothesized that these changes would be more evident in cats with CDS.

## Materials and Methods

### Sample Collection

The brains of cats of different ages were collected post-mortem through the routine necropsy service at The Royal (Dick) School of Veterinary Studies (RDSVS). Some of these cats were patients at the RDSVS Hospital for Small Animals, while others came from a rescue center in Scotland. Most of the cats were affected by old age and/or chronic disease and were euthanized after veterinary assessments revealed that further treatment would not benefit quality of life. A small number of cats were euthanized after being diagnosed with behavioral problems (e.g., untreatable aggression) that would impede their rehoming. In all cases, the owners or shelter manager gave written consent to donate the cats’ bodies for research purposes.

Ethical approval was obtained through the Veterinary Ethical Review Committee from the RDSVS, The University of Edinburgh (VERC 50.17 and 30.20).

Some of the patients had been diagnosed with CDS before euthanasia. For this diagnosis to be made, cats had to have displayed at least one behavioral change associated with CDS for at least three months (Gunn-Moore et al., 2007; Landsberg et al., 2010; Sordo and Gunn-Moore, 2021), and the behavioral changes could not be attributed to any other medical condition.

**FIGURE 1:**
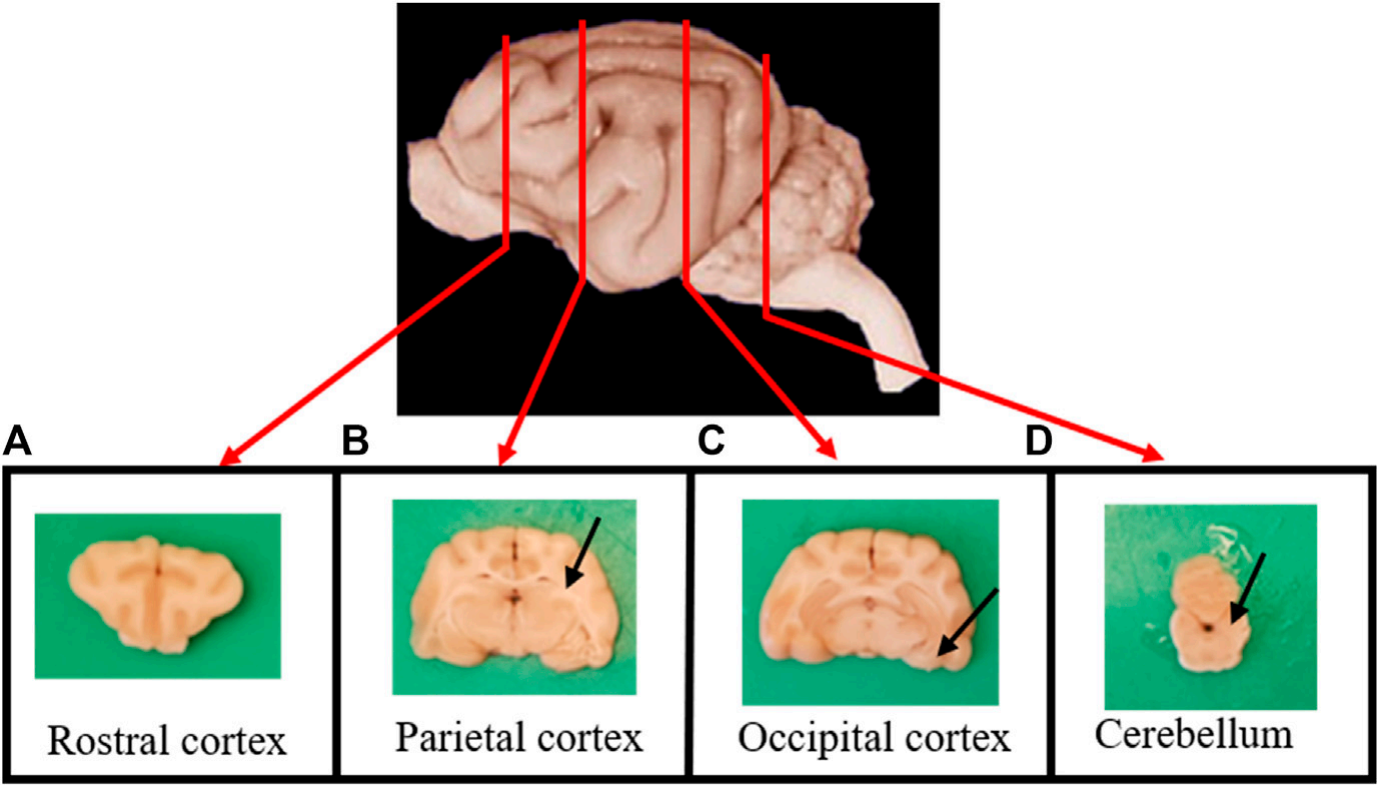
Transverse sections of the brain were taken from **(A)** the rostral cortex; **(B)** the parietal cortex, including the hippocampus (arrow); **(C)** the occipital cortex, including the entorhinal cortex (arrow); and **(D)** the cerebellum and the locus coeruleus (arrow).

After collection, brains were fixed by immersion in 10% buffered formalin for no less than one week. After fixation, transversal sections of the brain were taken from the rostral cortex (Figure 1A), parietal cortex, including hippocampus (Figure 1B), occipital cortex, including the entorhinal cortex (Figure 1C), the locus coeruleus and the cerebellum (Figure 1D).

Sections were processed overnight in paraffin (Leica Tissue Processor ASP300S; Leica Biosystems™) and blocks were made. Consecutive slices of 6 μm were taken from the blocks using a microtome (Microtome HM325; Leica Biosystems™) and mounted on positively charged glass slides (Polysine®, VWR™).

### Immunohistochemistry

Consecutive 6 μm sections were taken from the rostral, parietal (including hippocampus), and occipital (including entorhinal cortex) lobes, plus of the cerebellum and locus coeruleus. These regions are those that are typically affected by Aβ and hyperphosphorylated tau in the AD brain. After deparaffinization and rehydrating sections, antigen retrieval pre-treatment was performed by immersing slides in 90% formic acid for 6 h (Kraszpulski et al., 1998) for antibody 4G8, and heat incubation (120°C for 40 min) in sodium citrate buffer (10 mM, pH 6) for all other antibodies. All antibodies were incubated in 3% hydrogen peroxide in 10% methanol for 30 min to block endogenous peroxidase activity. Sections were incubated overnight at room temperature in each of the primary antibodies: two anti-*β*-amyloid and six anti-tau antibodies, listed in Table 1. Bound antibody was detected by incubating slides in biotinylated goat anti-mouse/rabbit antibodies (1:500; ThermoFisher Scientific™) for one hour, followed by incubation in ABC peroxidase kit (1:100; ThermoFisher Scientific™) for 30 min. Slides were visualized with 3, 3′-diaminobenzidine substrate (1:100; DAB; Sigma-Aldrich™) under light microscope (Nikon™ Brightfield). Negative controls were used by omitting the primary antibodies. Immunostaining was conducted while blind to the groupings of the animals.

**TABLE 1:** Primary antibodies for the detection of *ß*-amyloid and tau proteins in the cat brain.

Antigen	Clone	Dilution
Anti-β-amyloid 17–24 and APP (Biolegend™; San Diego, CA)	4G8	1:1,500
Anti-β-amyloid 1–42 (Abcam™; Cambridge, United Kingdom)	mOC64	1:6,000
Anti-tau 3-repeat isoform RD3 (Sigma-Aldrich™; Missouri, United States)	8E6/C11	1:1,000
Anti-tau 4-repeat isoform RD4 (Sigma-Aldrich™; Missouri, United States)	1E1/A6	1:1,000
Anti-tau 404–421 (Biolegend™; San Diego, CA	Tau46	1:1,000
Phospho-PHF-tau pSer202 + Thr205 (ThermoFisher Scientific™; Massachusetts, United States)	AT8	1:200
Purified anti-tau phospho (Ser 262; Biolegend™; San Diego, CA)	A15091A	1:1,000
Phospho-PHF-tau pThr212 + Ser214 (ThermoFisher Scientific™; Massachusetts, United States)	AT100	1:1,000

### Validation of Intranuclear Hyperphosphorylated tau Immunolabelling

#### Isotype Controls

To validate that intranuclear immunolabelling was not caused by artifact or non-specific staining, IHC was performed following the above protocol and using antibodies with the same isotypes for AT8 (mouse IgG isotype control; ThermoFisher Scientific™), AT100 (mouse IgG1 kappa isotype control; ThermoFisher Scientific™), and A15091A antibodies (mouse IgG2b kappa isotype control; Biolegend™) as negative controls.

#### Dephosphorylation of the tau Protein

To further validate that the intranuclear labelling observed in cat brains reflected the hyperphosphorylated state of the protein, as opposed to the non-phosphorylated protein, we used the approach of dephosphoryling the protein in brain samples. To achieve this, IHC was performed by following the protocol described above, for the same hyperphosphorylated tau antibodies (i.e., AT8, AT100, and A15091A), separately; however, to dephosphorylate the protein, slides were incubated in alkaline phosphatase (FastAP Thermosensitive Alkaline phosphatase; ThermoFisher Scientific™) following manufacturers’ protocol at 37°C for one hour, followed by three 5 min washes in fresh bovine serum albumin, before adding the primary antibodies using the protocol described in *Immunohistochemistry*.

#### Protein Extraction

Extracts from the cytoplasm, the cytoskeleton, the soluble nuclear fractions, and the chromatin bound nuclear fractions were obtained from frozen cat brain tissue (a 10-year-old cat) by using the Subcellular Protein Fractionation Kit for Tissues (ThermoFisher Scientific™) following manufacturer’s protocol.

#### Western Blot

Extracts from the different fractions (15 μl) obtained with the Subcellular Protein Fractionation Kit for Tissues (ThermoFisher Scientific™) were prepared in a buffer containing NuPAGE™ LDS Sample Buffer (4x) and NuPAGE™ MES running buffer (10x) and were loaded into a 12 well NuPAGE™ pre-cast gel, 4–12% bis-tris. The gel ran at 50 V for 20 min, followed by 120 V for 20 min, and 200 V for 40 min. The proteins were then transferred onto an Immobilon-FL PVDF membrane (Millipore™) using a semi-dry electroblotting unit (Trans-Blot® SD semi-dry transfer cell; Bio-Rad™), run at 0.06 A and 18 V for one hour. The PVDF membrane was transferred into LICOR blocking buffer (Licor Biosciences™) for one hour. Primary antibodies were added (AT8, 1:1,000; and Tau46, 1:5,000), and blots were incubated at 4°C on a roller overnight. Goat anti-mouse antibodies were used as secondary antibodies (IRdye 680RD; Licor Biosciences™). Analysis was performed using LICOR odyssey (700 nm wavelength).

### Quantification of Protein Deposition

Initially, all of the slides were visually inspected by light microscopy; this included the slides from the rostral, parietal, and occipital regions, plus the cerebellum, that had been obtained after performing IHC with all the eight antibodies.

For the automated quantification of protein deposition, only the slides stained against mOC64 for *ß*-amyloid, and AT8 and AT100 for hyperphosphorylated tau, from all brain regions, were scanned using a Leica Aperio Versa 200 scanner (Leica Biosystems™).

Images were transferred to the Aperio eSlide Manager® v12.4.0.5043 and visualized with the Aperio ImageScope® v12.3.3.5048. Annotation boxes of 600 μm of width, height and length were manually placed for each brain region of interest; in total five annotation boxes were created for gray matter, and five for white matter; plus, three boxes for hippocampus, and three for cerebellum. The Positive Pixel Count v9 algorithm was used for the assessment of positive immunolabelling for the load and burden (see below) of Aβ and hyperphosphorylated tau. In addition, the Nuclear Algorithm v9 was used for the assessment of positive immunolabelling for intranuclear hyperphosphorylated tau.

Data from the Positive Pixel Count v9 algorithms were exported to an excel file, where the load (i.e., percentage of total positive pixels) and burden (i.e., the proportion of the total area assessed that contains positive labelling) of both *ß*-amyloid and hyperphosphorylated tau were calculated, by using the following equations:

$$Load\;of\;protein\;\left(\%\right)=\left(\frac{Nwp+Np+Nsp}{Ntotal}\right)x\;100$$ (1)

$$Burden\;of\;protein=\;\frac{\frac{Nwp+Np+Nsp}{Total\;area}}{10,000}\;\mathrm{pixels}/mm^{2}$$ (2)

where,

Nwp = Number of weak positives.

Np = Number of (moderate) positives.

Nsp = Number of strong positives.

Ntotal = Number of total positives and negatives.

Total area was 1.8 mm^2^ for cortical regions and 1.08 mm^2^ for cerebellum and hippocampus. These area measures were calculated by multiplying two dimensions of the annotation boxes (i.e., 600 μm × 600 μm), multiplying the result by the number of annotation boxes created for each region (i.e., multiply by five for cortical regions and by three for hippocampus and cerebellum) and converting the result to mm^2^.

Data obtained from the Nuclear Algorithm v9, including the total percentage of nuclei stained and the degree of staining (i.e., 1 +, 2 +, and 3 +) were exported to an excel sheet and used for statistical analyses.

### Data Analysis

Data were analyzed using Minitab® v19.2020.1 statistical software for Windows.

Anderson-Darling tests were performed to assess normality. Non-normal data were transformed using Box-Cox transformation or Johnson transformation. Bartlett tests for equal variances were performed with 95% confidence level (CI), assuming normal distribution. Non-parametric tests were performed in the variables in which transformation failed.

To determine whether there were differences in the positive immunolabelling of proteins (mOC64 for *ß*-amyloid, and AT8 and AT100 for hyperphosphorylated tau) in the different age groups, one-way ANOVA were performed assuming equal variances (if applicable) with two-sided 95% CI and a significance level of *a* = 0.05. Subsequently, we determined which group differences were significant using Tukey tests and Games-Howell post-hoc tests. Kruskal-Wallis tests were performed to assess differences in non-normal data.

To determine whether there were differences in the positive immunolabelling of the proteins between gray and white matter, 2-sample *t*-tests were performed with a 95% CI and a hypothesized difference of zero, assuming equal variances. Mann-Whitney tests were performed to assess differences in non-normal data.

To determine whether there were differences in the positive immunolabelling of the proteins between cats with and without CDS, 2-sample *t*-tests were performed as described above.

Finally, chi-square tests were performed to determine whether there was an association between cognitive status (i.e., cats with and without CDS) and the presence of pre-tangles.

## Results

### Demographics

A total of 55 cat brains were collected at necropsy. The majority of cats were non-pedigree (98.2%; *n* = 54), with only one cat being a pedigree breed (a Bengal cat). Half of the cats (52.7%; *n* = 29) were females; of these, two thirds (69%; *n* = 20) were neutered, and a third (31%; *n* = 9) were intact. A third of the cats (30.9%; *n* = 17) were males; most of them (88.3%; *n* = 15) were neutered and only 11.7% (*n* = 2) were intact. The sex of 16.4% (*n* = 9) of the cats was unknown, mainly because this was not recorded by the pathologists performing the brain extractions. Ages ranged between two and 25 years.

### Grouping of Cats

Since the precise age of some of the cats was uncertain, cats were grouped according to their age as Prime (three to six years old), Mature (seven to 10 years old), Senior (11–14 years old), and Super Senior (≥15 years old) (Vogt et al., 2017). After being grouped by age, six cats were in the Prime group; 26 in the Mature group; nine in the Senior group; and 14 in the Super Senior group. A total of ten cats had a confirmed diagnosis of CDS, eight of these cats were in the Super Senior group and two were in the Senior group. (Supplementary material 1).

### *β*-Amyloid Pathology

**FIGURE 2:**
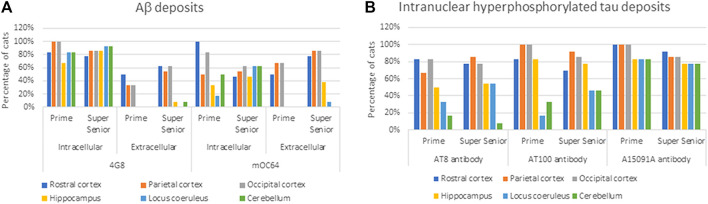
Percentage of cats from the Prime and Super Senior groups showing **(A)** intracytoplasmic and extracellular Aβ deposits in the different brain regions, with both 4G8 and mOC64 antibodies; and **(B)** intranuclear labeling of hyperphosphorylated tau (AT8, AT100, and A15091A antibodies) in the different brain regions.

Cats of all ages showed Aβ pathology (i.e., labeling with 4G8 and mOC64 antibodies). However, elderly cats (i.e., ≥15 years old) had more and larger extracellular Aβ deposits than younger cats. In contrast, younger cats had more positive immunolabelling for intracytoplasmic Aβ (Table 2) (Figure 2A).

**TABLE 2:** Percentage of cats from the Prime and Super Senior groups showing intracytoplasmic and extracellular Aβ deposits in the different brain regions, with both 4G8 and mOC64 antibodies.

	4G8 antibody				mOC64 antibody			
	Intracellular Aβ		Extracellular Aβ		Intracellular Aβ		Extracellular Aβ	
	Prime	Super senior	Prime	Super senior	Prime	Super senior	Prime	Super senior
Rostral cortex	83%	77%	50%	62%	100%	46%	50%	77%
Parietal cortex	100%	85%	33%	54%	50%	54%	67%	85%
Occipital cortex	100%	85%	33%	62%	83%	62%	67%	85%
Hippocampus	67%	85%	0	8%	33%	46%	0	38%
Locus coeruleus	83%	92%	0	0	17%	62%	0	8%
Cerebellum	83%	92%	0	8%	50%	62%	0	0

**FIGURE 3:**
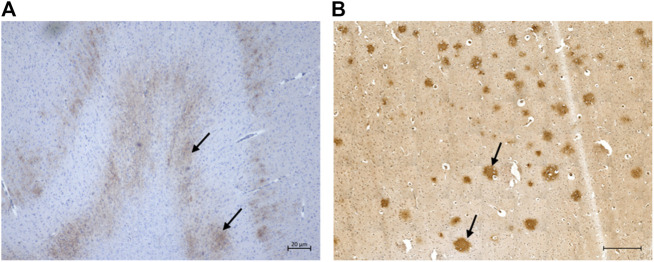
Extracellular *ß*-amyloid (Aβ) deposition in the cat brain. **(A)** Diffuse extracellular Aβ deposition in the parietal cortex of a 12-year-old cat (primary antibody mOC64), scale bar 20 μm; and **(B)** Extracellular Aβ plaque-like formation in the parietal cortex of a 16-year-old cat (primary antibody mOC64; scale bar 500 μm).

Extracellular Aβ deposition was mostly found in the cortex regions. These aggregates had a diffuse pattern (Figure 3A) which, in some cases, formed patches. Congo red and thioflavin-S staining were negative (data not shown). While most of the cats showed a diffuse Aβ distribution, one cat had multiple plaque-like formations (Figure 3B). Both the severity of pathology and the numbers of cats with extracellular Aβ deposits increased with age.

**FIGURE 4:**
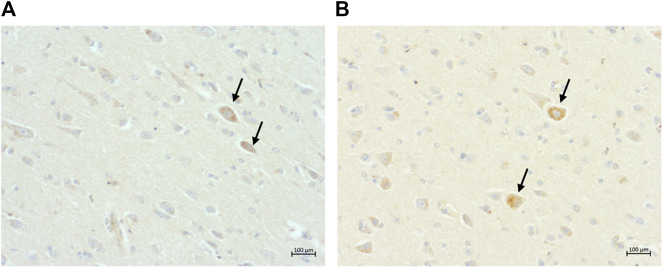
Intraneuronal intracytoplasmic immunolabelling of *ß*-amyloid in, **(A)** the parietal cortex of a 13-year-old cat (primary antibody 4G8); and **(B)** the occipital cortex of the same 13-year-old cat (primary antibody mOC64), scale bars 100 μm.

Intraneuronal intracytoplasmic Aβ immunolabelling was found in the younger cats (i.e., three to six years old; Figure 4) and the percentage of cats with these deposits decreased with age, particularly in cortex. However, the opposite was found in the hippocampus, locus coeruleus, and cerebellum, where the percentage of cats with intracytoplasmic Aβ immunolabelling increased with age (Figure 2A).

**FIGURE 5:**
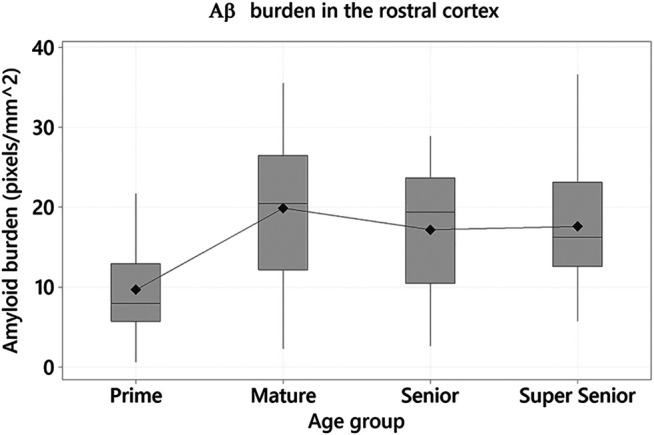
Amyloid-β (Aβ) burden in the rostral cortex of the cats in the different age groups. Cats in the Prime group had the lowest Aβ burden when compared to the Mature (*p* = 0.002) and Super Senior groups (*p* = 0.043).

One-way ANOVA were performed to determine whether age has an effect on Aβ pathology (mOC64 antibody) and to assess whether there were differences between the age groups. A statistically significant effect of age on Aβ burden was found in the rostral cortex (F_3_ = 4.55, *p* = 0.005). Tukey tests demonstrated the Prime group had the lowest positive immunolabelling, when compared to the other age groups. Statistically significant differences were found between the Prime and Mature groups (Difference of means = 10.24; SE of difference = 2.80; 95% CI [2.94, 17.54]; *t* = 3.66; *p* = 0.002) and between the Prime and Super Senior groups (Difference of means = 7.93; SE of difference = 2.97; 95% CI [0.17, 15.69]; *t* = 2.67; *p* = 0.043) (Figure 5).

When comparing the Aβ pathology between the different brain regions, the cerebellum had the lowest load of *ß*-amyloid when compared to the other regions (F_4_ = 4.58, *p* = 0.001).

Differences in Aβ pathology between gray and white matter were also assessed. Statistically significant differences in the positive Aβ labeling were found in all the cortex regions between gray and white matter, with the strongest immunolabelling being present in the gray matter (*p* < 0.001).

To determine whether the extent of Aβ deposits was greater in cats with CDS, we compared the extracellular Aβ load and burden in the different brain regions of cats with CDS with that in age-matched controls. However, 2-sample *t*-tests showed no statistically significant differences in the positive Aβ labeling between cats with and without CDS.

### Tau Pathology

**FIGURE 6:**
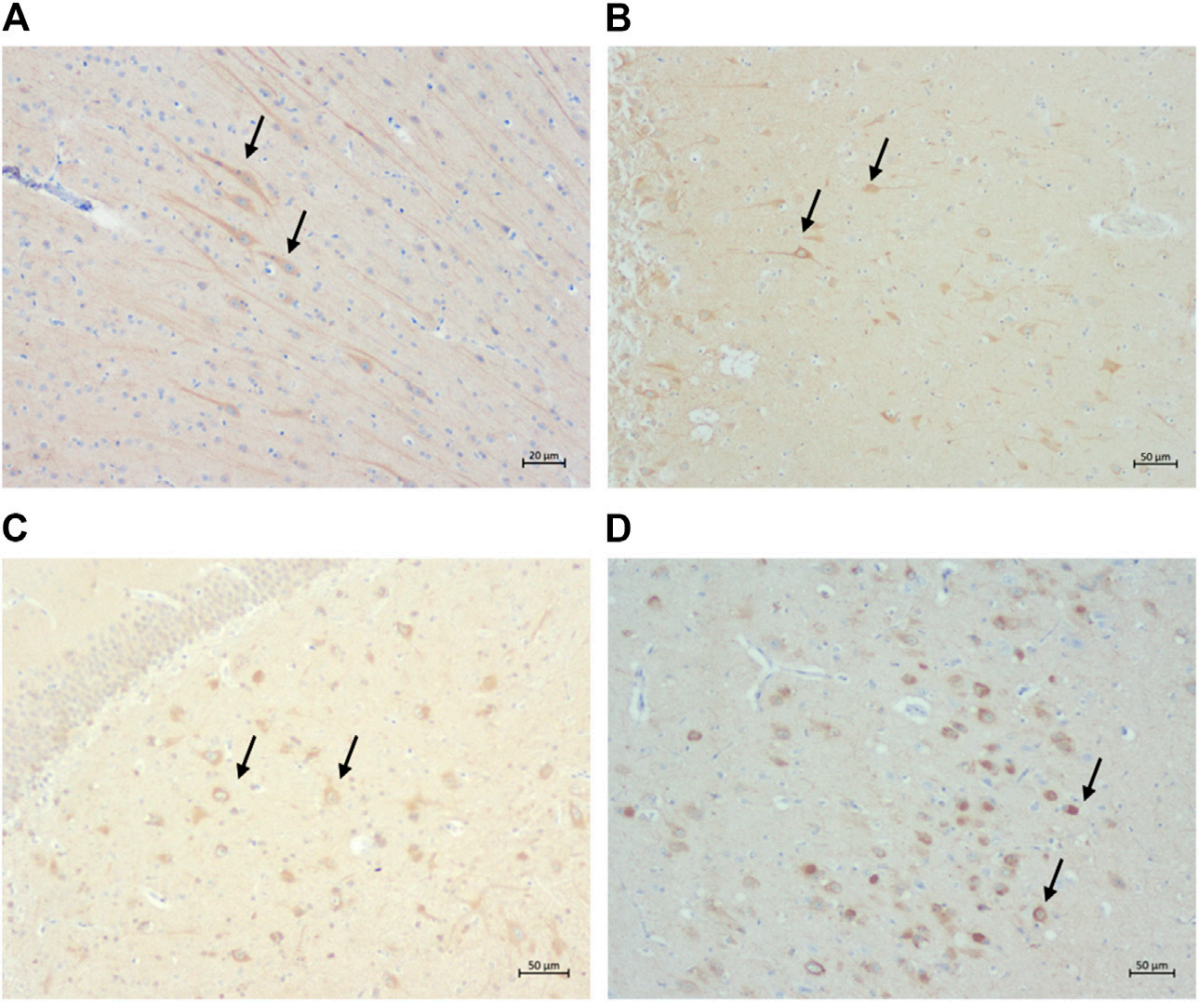
Intracytoplasmic immunolabelling of **(A)** total tau (primary antibody Tau46) in the rostral cortex of a 10-year-old cat, scale bar 20 μm; **(B)** total tau (primary antibody Tau46) in the parietal cortex of a 10-year-old cat, scale bar 50 μm; **(C)** 3-repeat tau isoform tau (primary antibody 8E6/C11) in the hippocampus of a 7-year-old cat, scale bar 50 μm; and **(D)** 4-repeat isoform of tau (primary antibody 1E1/A6) in the parietal cortex of a 19-year-old cat, scale bar 50 μm.

Intranuclear and intracytoplasmic immunolabelling of normal tau (i.e., Tau46 antibody) (Figures 6A,B) is found in cats of all ages and in all brain regions, as was intracytoplasmic immunolabelling of the two isoforms of tau: 3 and 4-repeat (i.e., labelled by 8E6/C11 and 1E1/A6 antibodies, respectively) (Figures 6C,D).

Intranuclear and intracytoplasmic immunolabelling for hyperphosphorylated tau (i.e., AT8, AT100, and A15091A antibodies) was also found within neurons. Intranuclear immunolabelling was primarily found in younger cats, whereas intracytoplasmic labelling (i.e., pre-tangles) was found most frequently in elderly cats (i.e., ≥15 years old).

**FIGURE 7:**
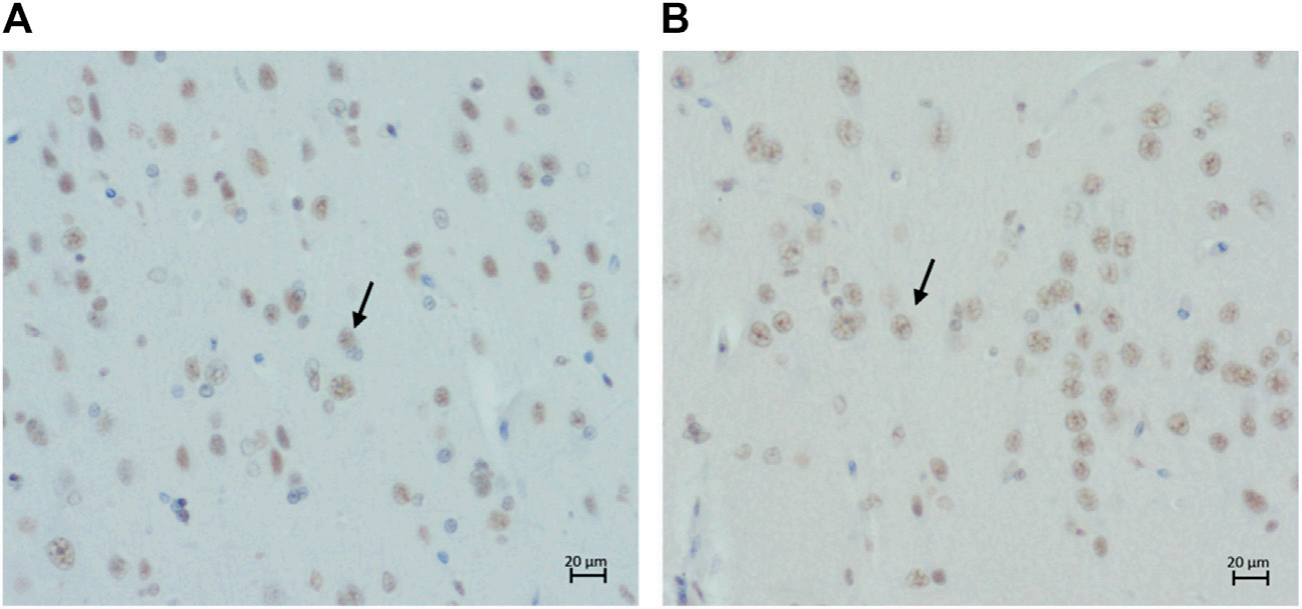
Intranuclear immunolabelling of hyperphosphorylated tau in **(A)** the parietal cortex of a 14-year-old cat (primary antibody AT8), scale bar 20 μm; and **(B)** the parietal cortex of a 14-years-old cat (primary antibody AT100), scale bar 20 μm.

Intranuclear immunolabelling of hyperphosphorylated tau was most commonly found in cortical regions of younger cats (i.e., three to six years old) and the proportion of cats showing positive immunolabelling tended to decrease with age (Figures 7A,B). In contrast, the percentage of cats showing intranuclear immunolabelling tended to increase with age in the locus coeruleus. However, these were not statistically significant (Table 3) (Figure 2B).

**TABLE 3:** Percentage of cats from the Prime and Super Senior groups showing intranuclear labeling of hyperphosphorylated tau (AT8, AT100, and A15091A antibodies) in neurons in the different brain regions.

Hyperphosphorylated tau						
	AT8 antibody		AT100 antibody		A15091A antibody	
	Prime (%)	Super senior (%)	Prime (%)	Super senior (%)	Prime (%)	Super senior (%)
Rostral cortex	83	77	83	69	100	92
Parietal cortex	67	85	100	92	100	85
Occipital cortex	83	77	100	85	100	85
Hippocampus	50	54	83	77	83	77
Locus coeruleus	33	54	17	46	83	77
Cerebellum	17	8	33	46	83	77

Intracytoplasmic immunolabelling for hyperphosphorylated tau (i.e., pre-tangles) was found within neurons with all three antibodies and its presence was confirmed by Silver-Gallyas staining (data not shown).

**FIGURE 8:**
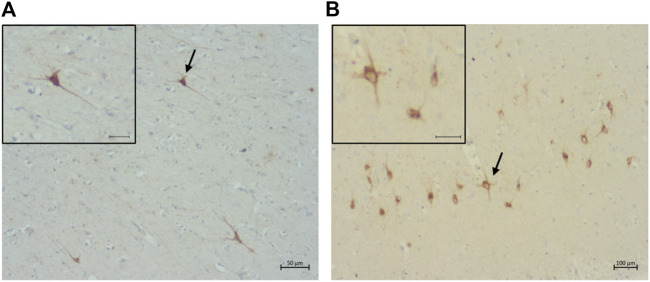
Pre-tangles of hyperphosphorylated tau in **(A)** the rostral cortex of a 10-year-old cat (primary antibody AT8), scale bar 50 μm; and **(B)** the occipital cortex of a 19-year-old (primary antibodyAT100), scale bar 100 μm.

A total of 14 cats were shown to have pre-tangles with the AT8 antibody (Figure 8A). Of these, one was in the Prime group, two in the Mature group, one in the Senior group, and ten in the Super Senior group. Similarly, 15 cats were shown to have pre-tangles with the AT100 antibody (Figure 8B), 14 of which also showed positive staining with AT8. The cat that showed labelling of pre-tangles with AT100 only was in the Prime group. Of note, the cats that had pre-tangles showed little to no intranuclear tau immunolabelling.

Six out of ten cats with a confirmed diagnosis of CDS had pre-tangles labelled by both AT8 and AT100 mostly in the cerebral cortex, and an additional cat with CDS had a similar distribution of pre-tangles that were labelled by AT100 only. In contrast, labeling of pre-tangles with the A15091A antibody, which binds to phosphorylated Ser 262 in human tau, was found in only three cats, one in the Mature group, and two in the Super Senior group. One of the positive cats from the Super Senior group had a confirmed diagnosis of CDS, and its pre-tangles were also detected using AT8 and AT100 antibodies.

**FIGURE 9:**
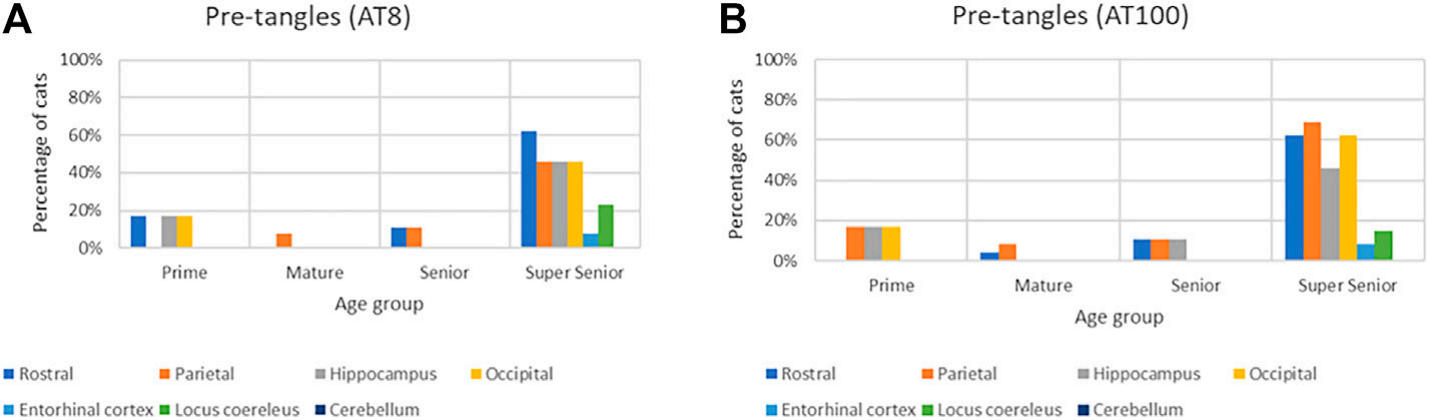
Association of the presence of pre-tangles with age in different brain regions with both AT8 **(A)** and AT100 **(B)** antibodies.

The proportion of cats in which pre-tangles were detected with both AT8 and AT100 antibodies increased in all of the brain regions in the Super Senior group (Figure 9).

**FIGURE 10:**
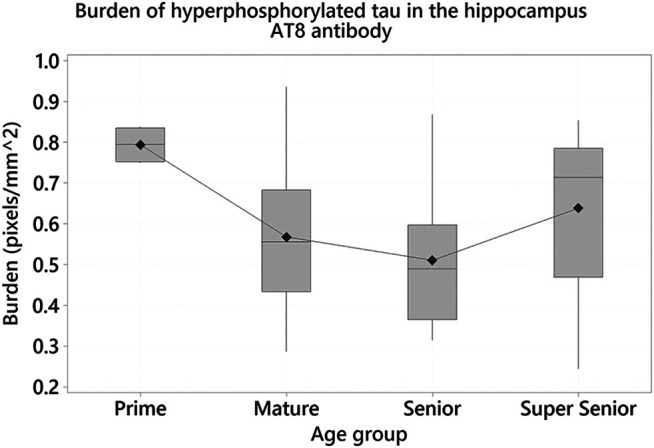
Cats in the Prime group had a higher burden of hyperphosphorylated tau with AT8 antibody in the hippocampus when compared to the Senior group (*p* = 0.038).

One-way ANOVA showed statistically significant differences in the burden of hyperphosphorylated tau with the AT8 antibody in the hippocampus (F_3_ = 3.08, *p* = 0.036). Tukey tests demonstrated that the Prime group had a higher burden, when compared to other age groups. Statistically significant differences were found between the Prime and Senior groups (Difference of means = –0.284; SE of difference = 0.102; 95% CI [–0.556, –0.012]; *t* = –2.77; *p* = 0.038) (Figure 10).

Two-sample t-tests showed statistically significant differences in the loads of hyperphosphorylated tau between gray and white matter; higher loads were found in the gray matter of the rostral (*t* = 2.12, *p* = 0.036) and occipital (*t* = 2.14, *p* = 0.035) cortices, with AT8 antibody; and in the parietal (*t* = 2.58, *p* = 0.011) and occipital (*t* = 2.73, *p* = 0.007) cortices, with AT100 antibody.

The largest numbers of pre-tangles were found in cats with CDS. Most of these cats (6/10) were shown to have pre-tangles with both the AT8 and AT100 antibodies in the rostral cortex (X^2^ = 31.534, *p* < 0.001; and X^2^ = 35.224, *p* < 0.001, respectively), the parietal cortex (X^2^ = 11.6, *p* = 0.001; and X^2^ = 19.512, *p* < 0.001, respectively), the occipital cortex (X^2^ = 22.25, *p* < 0.001; and X^2^ = 32.245, *p* < 0.001, respectively), and the hippocampus (X^2^ = 8.709, *p* = 0.003; and X^2^ = 11.583, *p* = 0.001, respectively) when compared with age-matched controls.

**FIGURE 11:**
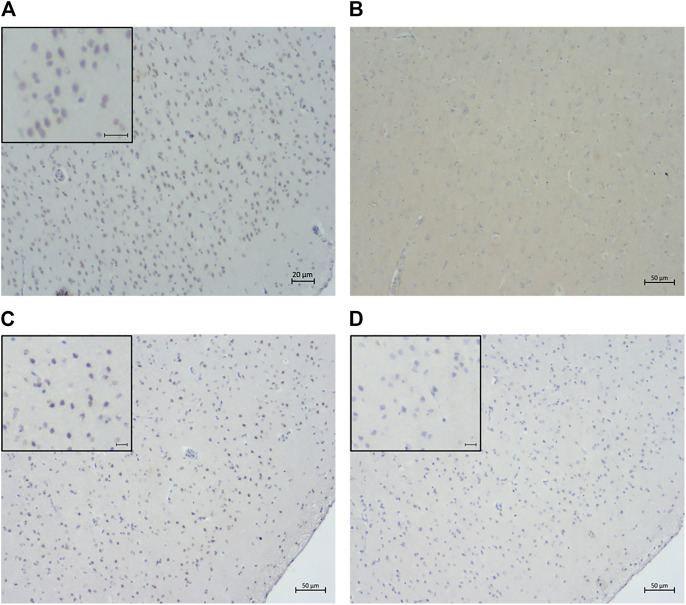
Confirmation of the specificity of intranuclear immunolabeling for hyperphosphorylated tau. Sections of rostral cerebral cortex from a 14-year-old cat were labeled by IHC using either AT8 **(A)** or an isotype control **(B)** as primary antibody. Intranuclear staining was not seen in the isotype control section **(B)**. Sections of rostral cerebral cortex from an 18-year-old cat were labeled with AT8 either without **(C)** or following alkaline phosphatase (AP) treatment **(D)**. No positive intranuclear staining was seen in the AP treated section **(D)**. Scale bars = 20 μm in **(A)** and 50 μm in **(B)**, **(C)**, and **(D)**.

It could be argued that the intranuclear hyperphosphorylated tau may be a consequence of nonspecific binding of the antibody to normal tau, hence, a number of controls were included. To confirm that intranuclear immunolabelling was not caused by artifact or non-specific staining, IHC was performed using antibodies with irrelevant specificities but the same isotypes as AT8, AT100, and A15091A antibodies as negative controls (Figures 11A,B). To validate that the intranuclear labelling was detecting hyperphosphorylated tau, it was shown that in tissue sections dephosphorylated using alkaline phosphatase, intranuclear immunolabelling of tau was abolished (Figures 11C,D).

**FIGURE 12:**
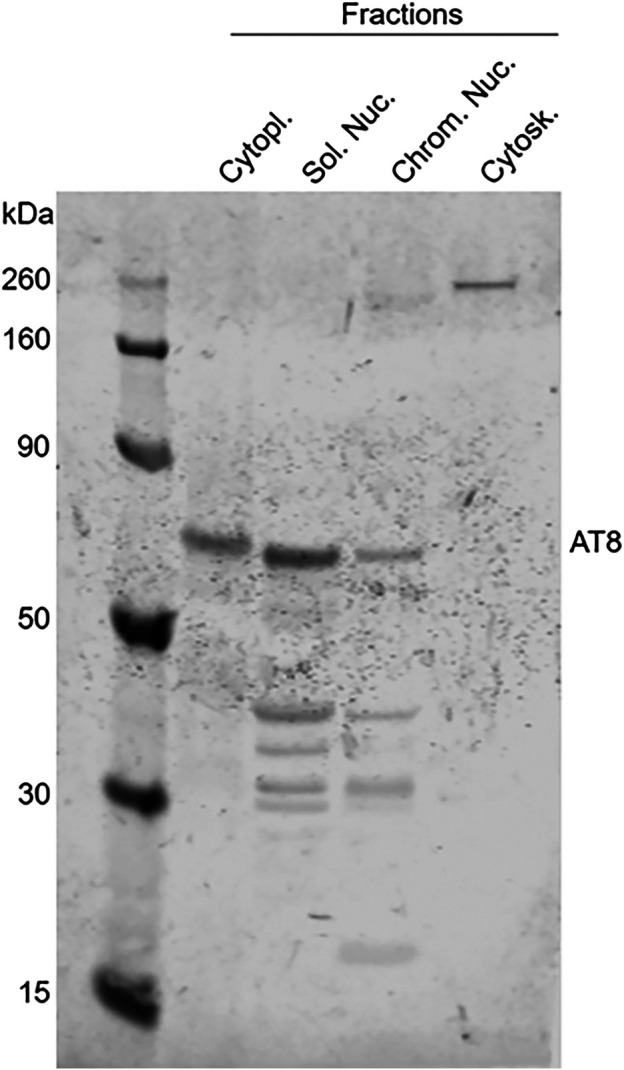
Detection of hyperphosphorylated tau in subcellular fractions extracted from the brain of a 10-year-old cat. Western blotting of different subcellular fractions, using AT8 as primary antibody, confirmed the presence of intranuclear hyperphosphorylated tau (AT8 antibody) in the soluble nuclear (Sol. Nuc.), chromatin-bound nuclear (Chrom. Nuc.) and cytoplasmic (Cytopl.) fractions but not in the cytoskeleton (Cytosk.).

Finally, a Western blot of different subcellular fractions extracted from a frozen cat brain (a 10-year-old cat) confirmed the presence of intranuclear hyperphosphorylated tau in both the soluble nuclear fraction and the chromatin bound fraction using the AT8 antibody, with a higher level of expression in the soluble nuclear fraction. Hyperphosphorylated tau was also found in the cytoplasm; however, it was not detected in the cytoskeletal fraction (Figure 12). A Western blot using an antibody against total tau (Tau46) gave similar results, although tau was also found in the cytoskeletal fraction with this antibody (data not shown).

## Discussion

This study shows that elderly cats develop both Aβ and tau pathologies; with large extracellular Aβ deposits and the presence of intracytoplasmic pre-tangles of hyperphosphorylated tau within neurons. Of note, the largest numbers of pre-tangles were found in the brains of cats with CDS. In contrast, younger cats generally had more intracytoplasmic Aβ deposits within neurons, and no pre-tangles; however, they did have higher intranuclear tau labelling, unlike the older cats.

### Aβ Pathology in the Cat Brain and Its Similarities to AD

Extracellular deposits of Aβ were found in the brains of most of the cats in this study. While there was some variability between the individual brains, which also occurs in humans (Braak and Braak, 1991; Thal et al., 2002), a similar distribution pattern was observed in most of them (see below).

Cats of all ages had extracellular Aβ deposits; however, the extent of these deposits varied between age groups. Smaller deposits were found in younger cats, whereas the largest deposits were found in cats of the Mature and Super Senior group, particularly in the rostral cortex. Despite cats in the Prime group having smaller Aβ deposits than cats in the Senior group, this was not statistically significant. While this could be due to the lack of power, it is also possible that Aβ accumulation suddenly increases, reaching high levels when cats are between 7–10 years old (Mature group) and then slowly progresses over time.

In the current study, the youngest cat with extracellular Aβ deposits was four years old; this contradicts previous studies that only reported Aβ deposits in cats over eight years of age (Cummings et al., 1996; Head et al., 2005; Gunn-Moore et al., 2006; Chambers et al., 2015). Extracellular Aβ deposition in cats in the Prime group was only found in the cortical areas (mainly rostral cortex with 4G8 labelling, and parietal and occipital cortices with mOC64). That Aβ deposits start accumulating in cortical regions in younger cat brains is perhaps not surprising. According to Braak and Braak, these are the same regions where Aβ deposition starts in the human brain (i.e., Stage I) (Braak and Braak, 1991; Thal et al., 2002). In humans, Aβ can begin accumulating in individuals as young as 20-years-old, based upon *in vivo* PET imaging, which is decades before developing any type of cognitive decline or dementia (Gonneaud et al., 2017). According to International Cat Care and their equivalence table between human and cat ages, a cat of four years old correspond to a human of ∼32 years old (Vogt et al., 2017), so this correlates well with when Aβ starts accumulating in humans.

Extracellular Aβ deposits were only found in the hippocampus in cats in the Super Senior group. Since cats in this age group also had larger deposits of Aβ in all the cortical brain regions, it is perhaps logical to conclude that, after accumulating in the cortical areas, Aβ pathology in cats progresses to the hippocampus. This same pattern has been described in the human brain during Stage II of Aβ progression (Braak and Braak, 1991; Thal et al., 2002). However, more studies are needed to confirm this pattern of progression in cats.

While the cortical regions and the hippocampus were the regions most affected by extracellular Aβ deposition in cats, the cerebellum was least affected. In humans, the cerebellum is only affected during the final stage of Aβ progression (i.e., Stage V) (Braak and Braak, 1991; Thal et al., 2002). Hence, it is possible that these cats did not live long enough to allow progression of Aβ deposition to the cerebellum.

Larger extracellular Aβ deposits were found affecting gray matter, when compared to white matter; this was the same in all cortical regions. This also correlates to the Aβ progression proposed by Braak and Braak, as gray matter is initially affected during Stage I, progressing to the white matter during Stage II of disease (Braak and Braak, 1991).

As has been shown previously, extracellular Aβ deposits in cats have a diffuse pattern (Head et al., 2005; Gunn-Moore et al., 2006; Chambers et al., 2015). This may explain why they were not detected with Congo red or thioflavin-S staining. However, in some cases, these diffuse deposits did form denser patches. This distribution pattern, including the occasional formation of patches, has been previously documented in the cat brain (Head et al., 2005; Gunn-Moore et al., 2006; Chambers et al., 2015). Of note, these deposits only contain the Aβ42 peptide (Cummings et al., 1996; Head et al., 2005).

Diffuse Aβ deposits in humans are mainly formed from this same peptide (Aβ42); these are believed to be an early stage of plaques (Hardy and Allsop, 1991; Perl, 2010). Interestingly, one 16-year-old cat in the current study had accumulations of Aβ in well-defined plaque-like structures. This may be a sporadic occurrence, which is supported by finding that the oldest cat in this study (a 25-year-old-cat) had diffuse Aβ deposition but no plaque-like structures. However, it is also possible that while some cats can form more compact senile plaques, few of them live long enough to do so. Further studies are required to confirm this hypothesis.

No statistically significant difference was found in the presence and/or extent of Aβ pathology between cats with and without CDS. This may be due to the low numbers of cats with a confirmed diagnosis of CDS. It is also plausible that Aβ pathology in cats does not correlate to the behavioral changes related to CDS, as previously suggested (Head et al., 2005). In humans, there is still contradictory evidence regarding the correlation between Aβ pathology and cognitive impairment. It is true that some studies have found a correlation between Aβ deposition and cognitive decline (Blessed et al., 1968). However, these studies have some caveats; for instance, they used silver stains that do not differentiate between senile plaques and diffuse deposits. In contrast, there is mounting evidence showing that Aβ pathology does not correlate with cognitive decline; moreover, there have been individuals with significant Aβ accumulation and no signs of cognitive impairment (Braak and Braak, 1996; Aizenstein et al., 2008; Villemagne et al., 2011; Ricciarelli and Fedele, 2017).

In humans, Aβ deposits accumulate within the cytoplasm of cells, particularly neurons, as well as in the extracellular spaces.

Intracytoplasmic Aβ deposition is believed to begin at a young age and to progress with age (Gouras et al., 2000; Baker-Nigh et al., 2015). For this reason, it has been suggested that intracellular Aβ has a normal function, potentially as an antioxidant (Smith et al., 2002; Nunomura et al., 2010). During early stages, intracytoplasmic Aβ is expressed at low concentrations and does not produce significant damage (Volloch et al., 2020); however, as individuals get older, it continues to accumulate, until it reaches higher concentrations and becomes toxic (Puzzo and Arancio, 2013). High concentrations of intracytoplasmic Aβ are linked to mitochondrial and cytoskeleton dysfunction, plus synaptic alterations (Gouras et al., 2000; LaFerla et al., 2007; Volloch et al., 2020). Furthermore, once a neuron dies, Aβ is released into the extracellular space, where it may promote further Aβ accumulation (LaFerla et al., 2007). Not surprisingly, correlations between the amount of intra and extracellular Aβ have been reported, as intracellular Aβ concentrations tend to decrease when extracellular deposits accumulate (Mori et al., 2002; Baker-Nigh et al., 2015).

In the present study, cats of all ages show evidence of intracytoplasmic Aβ deposition, predominantly within neurons in cortical areas. When assessing these areas, the proportion of cats with intracellular immunolabelling was highest in the Prime group, and it tended to decrease with age, especially where large extracellular Aβ deposits were found. However, in other areas, such as the hippocampus, locus coeruleus, and cerebellum, the proportion of cats with intracytoplasmic Aβ labelling increased with age. It could be argued that this positive intracytoplasmic labelling may represent APP, rather than Aβ, as the 4G8 antibody binds to the residues 17–24 of Aβ, plus APP; however, intracytoplasmic labelling was also found with the mOC64 antibody, which is a conformation-specific antibody that recognizes monomers, oligomers, and fibrils of Aβ42. Furthermore, thioflavin-S staining confirmed the amyloid structure. As is the case with humans (as discussed above), these findings suggest that intracytoplasmic Aβ starts accumulating in cortical areas of the cat brain at a young age, but then decreases in these regions as cats get older and extracellular Aβ accumulation increases. Longitudinal studies are needed to determine the progression of these deposits both within the cells and in the extracellular spaces, in addition to looking at the correlation between them. However, given the current lack of a suitable *in vivo* methods of doing this, it may be possible to assess Aβ in the cerebrospinal fluid of cats to determine the progression of Aβ pathology, which could be later correlated with the neuropathology.

### Tau Pathology in the Cat Brain and Its Similarities to AD

The current study found that non-phosphorylated tau protein was present in the brains of cats of all ages using all three tau antibodies. One of the antibodies, Tau46, binds to the C-terminus of the protein, while the other two (8E6/C11 and 1E1/A6) bind to the three and four repeats isoforms of the protein, respectively. Labelling with these three antibodies showed that tau was highly expressed in all of the brain regions, in all of the cats at all ages, as expected. This correlates with the expression of this protein and its isoforms in the adult human brain (Goedert et al., 1989; Šimić et al., 2016).

Hyperphosphorylated tau was detected within the cytoplasm of neurons in some of the cats, using all three antibodies against hyperphosphorylated tau (i.e., AT8, AT100, and A15091A). Since these aggregates tended to occupy the whole cytoplasm and yet appeared in neurons with normal morphology, they are believed to be pre-tangles (Braak and Del Tredici, 2010), which have been reported previously in cat brains (Head et al., 2005; Gunn-Moore et al., 2006; Chambers et al., 2015).

Pre-tangles were primarily observed in the cortex of cats in the Super Senior group, suggesting that significant tau pathology starts in cortical regions of the cat brain. If true, this may represent a difference in the onset of tau pathology between cats and humans, as it is known to start in the transentorhinal cortex in the latter (Braak and Braak, 1991). However, since the transentorhinal cortex was not assessed in the present study, it is impossible to determine whether tau pathology truly begins in the cortical regions or in the transentorhinal cortex in this species. In addition to the cortex, one of the cats in this study, diagnosed with CDS had small numbers of pre-tangles within their entorhinal cortex.

The load of pre-tangles varied between gray and white matter. The largest loads of pre-tangles were noted in the rostral and occipital cortices in gray matter.

Positive immunolabelling for hyperphosphorylated tau was also found in the hippocampus, where statistically significant differences were found in the burden between the Prime and Senior groups, with a higher burden seen in the Prime group, which was unexpected. However, we noted that the ImageScope image analysis software cannot distinguish nuclear from cytoplasmic labeling. If this is true, it is possible that the positive staining seen in the Prime group corresponds to intranuclear staining, rather than pre-tangles; after visual inspection of the slides this was found to be the case, with the intranuclear staining being stronger in the cats in the Prime group.

Interestingly, few pre-tangles were found in the cortex of two cats in the Prime group (i.e., one 4-year-old cat and one 6-year-old cat). Of note, this 4-year-old cat was the same cat that had extracellular deposition of Aβ. While finding pre-tangles in such young cats was unexpected, it was not entirely surprising. It is possible that these cats had undiagnosed neurological disorder that produced tauopathy, or that their age was miscalculated (which is not uncommon in cats that come from a rescue shelter), and they were in fact older than recorded. However, it is also plausible that tau pathology starts at a young age in some cats. Braak suggested that the early stages of tau pathology may occur in younger individuals, as NFT have been found in people under 25 years of age (Braak and Del Tredici, 2010); however, as individuals get older, the number of NFT increases, leading to more severe stages of disease (Braak and Del Tredici, 2010).

While no statistically significant differences were found in either the load or the burden of pre-tangles between cats with and without CDS, associations were found between the presence of pre-tangles and CDS; this is nuanced, but important. Finding an association between the presence of pre-tangles and CDS is not surprising; half of the cats shown to have pre-tangles had a confirmed diagnosis of CDS. This was supported by Chi-square tests that showed that cats with CDS had larger numbers of pre-tangles, compared to cats without CDS. In humans, a strong association has been reported between the presence of NFT and cognitive impairment (Nelson et al., 2012). Furthermore, the progression stages of tau pathology have been associated with different stages of cognitive impairment and dementia. While cognition is known to remain unaffected during Stages I and II, a mild cognitive impairment has been associated to Stages III and IV and, finally, AD is associated with the most severe stages of pathology (i.e., Stages V and VI) (Braak and Del Tredici, 2010).

While there is a plethora of evidence describing the involvement of NFT in the development of neurodegeneration and AD, recent studies have shown that these insoluble tau aggregates are not as toxic as it was originally proposed (Mi and Johnson, 2006; Mroczko et al., 2019). Instead, it has been suggested that either monomers or soluble oligomers with abnormal post-translational modifications, such as acetylation and increased phosphorylation at specific residues (e.g., acetylation of lysines K174 and K281; and phosphorylation of Thr231 and Tyr18) play a vital role in causing neuronal dysfunction (Irwin et al., 2012; Tracy and Gan, 2017; Neddens et al., 2018; Guha et al., 2020). The role of tau in the development of AD and cognitive decline remains unclear. On one hand, tau is believed to be the cause and potential trigger of disease, on the other, more recent evidence proposes that it plays a protective role instead (see below).

The presence of intranuclear was first reported in the early nineties in human neuroblastoma (Loomis et al., 1990; Greenwood and Johnson, 1995) and other cells, such as fibroblasts and lymphocytes (Thurston et al., 1996). Of note, one of these studies found that around 16% of the total tau protein is located within the nucleus, particularly in the chromatin (Greenwood and Johnson, 1995).

In the present study, in addition to IHC labelling of intranuclear tau, Western blots demonstrated that both hyperphosphorylated and non-phosphorylated states of tau were present in the nuclear fraction of cat brain extracts. In humans, both states of intranuclear tau are also known to exist and are believed to have different functions (Greenwood and Johnson, 1995; Rady et al., 1995).

Studies in rodents have shown that nuclear non-phosphorylated tau binds and protects DNA after induced oxidative stress or heat-shock (Papasozomenos and Su, 1991; Sultan et al., 2011; Maina et al., 2016). These two processes have been shown to promote the translocation and accumulation of non-phosphorylated tau into the nucleus and nucleolus in order to protect and maintain the integrity of DNA (Papasozomenos and Su, 1991; Sultan et al., 2011). These studies have shown that there is a significant dephosphorylation of cytoplasmic tau and an increase in nuclear tau immediately after inducing stress; whereas the opposite was observed after 24 h, with an increase in cytoplasmic tau phosphorylation and a decrease in nuclear tau (Sultan et al., 2011). Because of this, it has been suggested that phosphorylation may regulate the translocation of tau between the nucleus and the cytoplasm (Sultan et al., 2011). Furthermore, abnormal phosphorylation may alter the ability of tau to translocate and/or may affect its ability to bind to DNA and protect it from damage (Lefebvre et al., 2003; Sultan et al., 2011; Qi et al., 2015; Ulrich et al., 2018).

Nuclear tau is reported in the brains of AD patients, however, its role in the development of disease remains unclear (Maina et al., 2016). Following the amyloid cascade hypothesis, Aβ deposits may promote abnormal phosphorylation of tau, altering its translocation to the nucleus, and impeding its binding and protection of DNA (Maina et al., 2016), as described above.

In the present study, hyperphosphorylated intranuclear tau was primarily found within the cortical neurons of the cats. However, while no statistically significant differences were found between the different age groups, cats in the Prime group tended to have more hyperphosphorylated intranuclear tau, compared to the older age groups. The opposite was seen with in the cats that had pre-tangles, which had little or no intranuclear tau. In contrast, studies in human brains show that nuclear tau increases with age, reaching the highest levels at geriatric stages (Gil et al., 2017); however, in AD, intranuclear tau significantly decreases in the hippocampus and cortex, completely disappearing in advanced stages of disease (Hernández-Ortega et al., 2016).

There are a number of limitations to the present study. Since the neuropathology was only assessed post-mortem, the findings showed in this study represent a snapshot of the neuropathology of aging and CDS in cats. Further studies are needed to determine at what age these changes begin to occur and how the disease progresses over time. Furthermore, since neither behavioral changes nor cognition were formally assessed in most of the cats, no correlations can be evaluated between these and the presence and/or severity of the neuropathology.

Another limitation arose when trying to trace and retrieve the cats’ information, particularly where brains were collected several years before the study started; no information was recorded for some of the cats (i.e., sex, exact age, or medical record). Having this information would have helped to understand and/or explain the neuropathological changes observed in these cats, especially in the younger ones.

A limitation concerning the sectioning of the brains was also identified. All brains were manually sectioned by one of the authors (LS) without using a brain slicer, as had been developed for rats’ and mice’ brains. This resulted in slight variations in the thickness of the brain slices. In addition, there were natural individual variation in both the size and the shape of the brains. Even though anatomical landmarks were used to minimize variation when slicing the brains, there were differences in the size of some of the structures such as the hippocampus and cerebellum. This impeded analyzing these regions in more detail; for example, since the hippocampus was not sectioned in the exact same level for all the cats, assessing differences between the regions within the hippocampus could not be assessed.

Finally, the inability of the Positive Pixel algorithm to differentiate between nuclear or cytoplasmic immunolabelling represents a limitation. Since all the slides were visually inspected before being scanned, this issue was identified promptly. As a result of this limitation, and to examine the nuclear immunolabelling in more detail, the Nuclear algorithm was used in addition to the Positive Pixel quantification.

In summary, and despite the limitations noted above, the current study is the largest yet to examine the brains of cats of various ages for the presence or absence of Aβ and hyperphosphorylated tau deposits. This study found that cats accumulate Aβ as intracytoplasmic deposits and diffuse extracellular deposits; in addition, we found hyperphosphorylated tau within neurons, either as intranuclear or intracytoplasmic deposits, with the cytoplasm representing pre-tangles. The extent of extracellular Aβ deposits and the number of pre-tangles increase with age, as does the percentage of cats with pre-tangles. Finally, the presence and progression of these pathologies in terms of their brain location are similar to those seen in humans with AD, suggesting that the cat has the potential to be a natural model for the study of this condition.

## Data Availability

The raw data supporting the conclusions of this article will be made available by the authors, without undue reservation.
