# Supplementary material for: Neuropathology of Aging in Cats and its Similarities to Human Alzheimer’s Disease
Source: Front Aging. 2021 Jun 7;2:684607. doi: 10.3389/fragi.2021.684607 (PMC9261448; doi:10.3389/fragi.2021.684607)
Supplement: Supplementary file 1 [file Table1.DOCX]

*Cats were grouped according to their age as Prime (three to six years old), Mature (seven to 10 years old), Senior (11 to 14 years old), and Super Senior (≥ 15 years old)*

|  | **Cat** | **Age** | **Sex** | **CDS** |
| --- | --- | --- | --- | --- |
| **Senior** | 33 | 14 | FN | Yes |
|  | 34 | 13 | MN | No |
|  | 35 | 12 | FN | No |
|  | 36 | 13 | M | No |
|  | 37 | 12 | F | No |
|  | 38 | 14 | MN | No |
|  | 39 | 12 | FN | No |
|  | 40 | 11 | FN | Yes |
| **Super Senior** | 41 | 14 | M | No |
|  | 42 | 18 | FN | Yes |
|  | 43 | 20 | MN | No |
|  | 44 | 19 | FN | No |
|  | 45 | 15 | FN | No |
|  | 46 | 16 | MN | No |
|  | 47 | 19 | MN | Yes |
|  | 48 | 25 | FN | Yes |
|  | 49 | 16 | FN | Yes |
|  | 50 | 17 | FN | No |
|  | 51 | 20 | FN | Yes |
|  | 52 | 19 | FN | Yes |
|  | 53 | 19 | MN | Yes |
|  | 54 | 16 | F | No |
|  | 55 | 16 | FN | Yes |

|  | **Cat** | **Age** | **Sex** | **CDS** |
| --- | --- | --- | --- | --- |
| **Prime** | 1 | 6 | MN | No |
|  | 2 | 4 | - | No |
|  | 3 | 4 | FN | No |
|  | 4 | 4 | FN | No |
|  | 5 | 6 | FN | No |
|  | 6 | 2 | F | No |
| **Mature** | 7 | 10 | - | No |
|  | 8 | 10 | - | No |
|  | 9 | 10 | - | No |
|  | 10 | 10 | - | No |
|  | 11 | 10 | FN | No |
|  | 12 | 9 | MN | No |
|  | 13 | 10 | FN | No |
|  | 14 | 10 | - | No |
|  | 15 | 10 | - | No |
|  | 16 | 10 | - | No |
|  | 17 | 10 | MN | No |
|  | 18 | 10 | FN | No |
|  | 19 | 10 | MN | No |
|  | 20 | 9 | MN | No |
|  | 21 | 9 | MN | No |
|  | 22 | 10 | - | No |
|  | 23 | 10 | MN | No |
|  | 24 | 10 | FN | No |
|  | 25 | 10 | F | No |
|  | 26 | 9 | F | No |
|  | 27 | 8 | F | No |
|  | 28 | 9 | MN | No |
|  | 29 | 10 | MN | No |
|  | 30 | 10 | F | No |
|  | 31 | 10 | F | No |
|  | 32 | 7 | F | No |

*F = intact females, M = intact males, FN = female neutered, MN = male neutered*
